# A Machine Learning Approach for the NLP-Based Analysis of Cyber Threats and Vulnerabilities of the Healthcare Ecosystem †

**DOI:** 10.3390/s23020651

**Published:** 2023-01-06

**Authors:** Stefano Silvestri, Shareeful Islam, Spyridon Papastergiou, Christos Tzagkarakis, Mario Ciampi

**Affiliations:** 1 Institute for High Performance Computing and Networking, National Research Council of Italy (ICAR-CNR), Via Pietro Castellino 111, 80131 Naples, Italy; 2 School of Computing and Information Science, Anglia Ruskin University, Cambridge CB1 1PT, UK; 3 Focal Point, 1410 Waterloo, Belgium; 4 Department of Informatics, University of Piraeus, GR-185 34 Piraeus, Greece; 5 Institute of Computer Science, Foundation for Research and Technology-Hellas, GR-700 13 Heraklion, Greece

**Keywords:** healthcare ecosystem, cyber threats, cyber vulnerabilities, healthcare information infrastructure, natural language processing, machine learning

## Abstract

Digitization in healthcare systems, with the wid adoption of Electronic Health Records, connected medical devices, software and systems providing efficient healthcare service delivery and management. On the other hand, the use of these systems has significantly increased cyber threats in the healthcare sector. Vulnerabilities in the existing and legacy systems are one of the key causes for the threats and related risks. Understanding and addressing the threats from the connected medical devices and other parts of the ICT health infrastructure are of paramount importance for ensuring security within the overall healthcare ecosystem. Threat and vulnerability analysis provides an effective way to lower the impact of risks relating to the existing vulnerabilities. However, this is a challenging task due to the availability of massive data which makes it difficult to identify potential patterns of security issues. This paper contributes towards an effective threats and vulnerabilities analysis by adopting Machine Learning models, such as the BERT neural language model and XGBoost, to extract updated information from the Natural Language documents largely available on the web, evaluating at the same time the level of the identified threats and vulnerabilities that can impact on the healthcare system, providing the required information for the most appropriate management of the risk. Experiments were performed based on CS news extracted from the Hacker News website and on Common Vulnerabilities and Exposures (CVE) vulnerability reports. The results demonstrate the effectiveness of the proposed approach, which provides a realistic manner to assess the threats and vulnerabilities from Natural Language texts, allowing adopting it in real-world Healthcare ecosystems.

## 1. Introduction

Digitization in the healthcare system provides many benefits including efficiency of the healthcare service delivery, cost-savings, patient safety and care quality. There is no doubt about the positive impact of digital transformation in the healthcare sector. However, despite these benefits, the adoption of digital technology provides many Cyber Security (CS) challenges that can pose any potential risks within the healthcare system [1]. This massive technological transformation increases the attack surface where threat actors can exploit possible threats for any potential risk within the Health Care Information Infrastructure (HCII). In recent years, several successful CS attacks were reported in the healthcare sector: nearly 90% of healthcare organizations experienced a data breach in 2018 [2]. There are significant numbers of connected devices within the healthcare system [3] and vulnerabilities within these connected devices can propagate to other parts of the network [4]. An example are flaws found in Braun’s infusion pump or Medtronic insulin pump, that could pose potential threat to the patient health [5], or simulated attacks realized to pacemakers and implantable cardiac defibrillators [6]. Medical Internet of Things (IoT) devices are currently considered critical vulnerabilities and sources of threats and risks in the healthcare domain. Furthermore, human factors have a crucial impact on the CS within Healthcare Organizations [7]. For these reasons, there is a need to understand the threats and vulnerabilities within the healthcare system so that control actions can be identified to ensure security of the system [8,9].

However, analyzing threats and vulnerabilities in the healthcare sectors is a challenging task, due to the large number of published vulnerabilities and the difficulty in identifying the text that relates with potential threats within a healthcare system context. The large amount of unstructured Natural Language (NL) Cyber Security (CS) data related to the healthcare domain is often freely available on the Internet. More in detail, this textual data contains crucial and updated information related to the assets of the Healthcare Information Infrastructure (HCII) including threats, vulnerabilities, attacks, and other important CS information, which could be very useful to improve the protection of the HCIIs. It is often difficult to identify and extract the relevant information from such kinds of texts, which are usually available on blog posts, CS news websites, social media and other similar sources. In particular, the complexity of the NL can present polysemy, irony, long sentences and other issues, in addition to the peculiarities the technical language used CS domain, which uses many non-standard abbreviations or acronyms [10]. Therefore, it is hard to define specific methodologies able to extract the required information buried under that huge amount of textual data. Mining and extracting the most updated CS threats and vulnerabilities from the huge amount of information available in NL documents on the Internet can support the establishment of situational awareness proactively monitoring and preventing CS issues [11], but specifically tailored approaches are required [12].

It is worth noting that some ethical issues could arise when using such kinds of information for research purposes [13]. Although on social media platforms there is an explicit agreement that informs the user that their data might be used by third-party companies and research institutions, in other cases, such as hacker forums, there is no explicit contract for informing the participants regarding the use of their data. According to [14], researchers need to deeply investigate ethical compliance even when the data seem to be public. Usually, in CS research the data are accessed and analyzed without the informed consent of participants, but acquiring informed consent could be practically impossible with datasets containing hundreds of data. In the case of the experimental assessment presented in this work, there is no personal data included, so there are no ethical issues.

Some of the issues related to the automatic processing of NL texts have been recently addressed in the literature, thanks to the definition of customized Machine Learning (ML) approaches that leverages the more recent Natural Language Processing (NLP) techniques applied to the CS domain [15,16,17,18]. In addition, it is possible to exploit some of the CS domain-specific Knowledge Bases (KBs) and catalogs [19]. A useful NLP task that can support the analysis and the Information Extraction (IE) from unstructured textual data is the Named Entity Extraction (NER). This task automatically extracts and classifies the named entities mentioned in a text, such as, in the case of the CS domain, attack types (e.g., *Denial of Service*, *fishing*, etc.), assets (*MySQL*, *Apache Tomcat*, etc.), threats (*ransomware*, etc.), vulnerabilities (*Broken Authentication*, *injection*, etc.), and others. Machine Learning-based algorithms, such as eXtreme Gradient Boosting [20], have been also successfully applied to classify textual documents in CS domain [21,22].

The implementation of end-to-end ML technologies for the identification, analysis and assessment of CS issues is still a challenging task, due to some implicit limits of ML, such as the need for large and trustable datasets, the lack of explainability of many models, and the computational power required [23]. Moreover, the deployment of ML-based systems in critical environments, such as the HCII, further complicates this scenario, due to the difficulties of acquiring and managing the data from the target environment and integrating the ML technologies within the existing HCII systems [24]. Our approach does not need to install any software in the existing HCII environment, nor does it need to acquire data from these systems, but it only requires a preliminary mapping of assets that composes the HCII, facilitating in this way the implementation and deployment phase.

This work addresses many of the aforementioned challenges by adopting ML models for the threat and vulnerability analysis of Natural Language text for securing HCII. The methodology proposed in this paper is part of a more complex individual risk assessment approach, developed among the activities of the EC-funded H2020 AI4HEALTHSEC project (https://www.ai4healthsec.eu (accessed on 30 October 2022)).

The main novelty introduced by the proposed approach is that NL documents are automatically processed by ML models not only to simply identify and classify CS threats and vulnerabilities, but also to assign them a corresponding severity level. Furthermore, the threats and vulnerabilities, with the corresponding levels, are mapped within the underlying assets of the HCII. In this way, it is possible to provide additional and updated information to improve the identification and management of the most appropriate actions required to mitigate the CS risks in the healthcare ecosystems.

In summary, this work makes three main contributions. First, threat and vulnerability assessments are considered from the overall healthcare system based on the underlying assets within the HCII. This makes it possible to understand assets and their dependencies within the system. Second, NL input sources, including CS news websites, CS blogs and social media, are used to identify and assess the threats vulnerabilities, extracting updated CS information from texts available on the web. Thirdly, the proposed approaches adopt ML-based NLP techniques (a BERT model fine-tuned on CS NER, logistic regression and XGBoost) not only to identify possible threats and vulnerabilities related to assets of HCIIs, but also to determine a level of the risk associated to a specific threat and vulnerability. Several experiments have been performed with different datasets for testing the threat and vulnerabilities assessment approaches.

The obtained results showed that the proposed methods allows identifying and, more important, automatically assigning a corresponding level to the threats and vulnerabilities based on the processing of NL documents available on the web. Therefore, it is possible to exploit these constantly updated resources, providing crucial information to identify the controlling actions to mitigate the associated risks.

The paper is structured as follows. The most recent related works are presented in Section 2. Then, Section 3 describes the details of the proposed methodology. The experimental assessment is presented in Section 4, also including the details of the used datasets and resources. Finally, Section 5 presents the conclusions and outlines possible future works.

## 2. Related Works

There are several works that focus on the threats and vulnerability analysis using several techniques. This section provides an overview of existing works which are relevant to our research. In particular, we examine the areas of threat modeling, attacks in the healthcare sector and ML-based threat analysis.

### 2.1. Threat Modeling and Cyber Attacks in the Healthcare Sector

Threat modeling is one of the key activities to understand the threats for system specific context and among the existing methods PASTA and Attack Tree are well known [25]. PASTA is a risk-centric approach that identifies security flaws and possible impact so that appropriate controls can be determined for the mitigation. The model advocates analyst-business collaboration with the intent to assess, document, and propose countermeasures relative to the likelihood of an attack. Attack Tree follows a tree-based hierarchical structure to describe security of a system. The root node considers the goal, while the lower level nodes consider the possible attack to the system. It provides potential attack patterns for specific targets while describing threats aimed at a system and the possible counterattack approaches to realize them.

The Centre for Internet Security (CIS) reveals that several attacks, such as ransomware, data breaches, DDoS, and inside threats are commonly used by the attacker in the healthcare sector [26]. A recent study showed that at least 20% of the medical device manufacturers experienced ransomware or malware attacks in the last 20 months [27]. Cyber attacks can target medical devices, such as infusion pumps and other medical devices [5,6], or healthcare services, such as medicine delivery of the healthcare system [28]. The works in the literature emphasize the control, such as patch management and incident management, to improve security of a hospital.

### 2.2. Threat and Vulnerability Analysis Using Machine Learning Models

There are several recent works that focus on threat and vulnerability detection and analysis based on Machine Learning (ML) models. In Ghaffarian et al. [29], a survey of ML and Data Mining techniques to mitigate the damages of software vulnerabilities is presented. The work identified four main categories for vulnerability prediction: (i) Prediction Models based on Software Metrics using supervised ML approaches; (ii) Anomaly Detection Approaches using unsupervised ML methods to automatically extract a model of normality or mine rules from the software source code, and detect vulnerabilities as deviant behavior from the normal majority; (iii) Vulnerable Code Pattern Recognition, based on supervised ML approaches to extract patterns of vulnerable code segments from many vulnerability code samples; and (iv) Miscellaneous Approaches, whose belong the other AI and ML-based approaches that cannot be categorized in the previous categories. The authors of [30] proposed a cyber supply chain threat analysis that integrates Random Forest and XGBoost algorithms for the threat prediction. The work considers threat intelligence and predicts the Tactics, Techniques, and Procedures (TTP) deployed for a cyber attack, demonstrating high accuracy in their experimental assessment. Another novel threat analysis framework was proposed by [31], SHChecker, combines ML and formal analysis capabilities for the Smart Healthcare Systems (SHSs). In detail, the paper focuses on Internet of Medical Things (IoMT) and adopts several ML algorithms, including Decision Tree (DT), Artificial Neural Network (ANN), K-means, and others. The results showed that in their experiments, the NN-based algorithms provide less accuracy than DT-based algorithms. The authors of one paper [32] presented a method to analyze the severity of CS threats analyzing the language of CS-related tweets through a DL approach. The experiments used a corpus of 6000 tweets containing the description of software vulnerabilities, annotated with the opinions of the authors toward their severity. The paper also presented a method for linking software vulnerabilities reported in tweets to CVEs and NVD KBs. The obtained results demonstrated a high-precision in forecasting high-severity vulnerabilities, also highlighting that reports of severe vulnerabilities extracted from online sources are predictive of real-world exploits. In Satyapanich et al. [33], a semantic schema to describe CS events was presented using Deep Learning-based Information Extraction (IE) pipeline to implement the automatic extraction of structured information about data breaches, ransomware and phishing attacks and the discovery and the patches of vulnerabilities. Threat intelligence within the cyber security domain provides a knowledge base for threat-related information and includes mechanism to present this this knowledge, such as taxonomies, sharing standards, and ontologies [19], which can be exploited to implement information extraction methods [34] specifically customised for the CS area.

Natural Language Processing (NLP) approaches have been widely considered for threat and vulnerability analysis. In Gao et al. [18], a data and knowledge-driven CS Named Entity Recognition (NER) method is presented, exploiting a Bidirectional Long Short Term Memory with Conditional Random Field (BiLSTM-CRF) architecture, including also a multi-head self-attention neural network with word embeddings trained on CS closed-domain texts to improve their effectiveness [35], in conjunction with KBs, for the recognition of the details of the assets (application, vendor, version, etc.) involved in CS issues. The authors of [15] presented an NLP DL-based architecture for the identification of relevant CS information, such as vulnerability exploitations, attack discoveries and advanced persistent threats. This architecture is composed of a word-embedding layer, a BiLSTM layer, and a CRF layer, concatenated with a further BiLSTM as output layer. The results of their experiments showed some improvements with respect to the baselines. In Nikoloudakis et al. [36], a ML-based situational awareness framework is presented which is able to detect existing and newly introduced network-enabled entities in an IoT-based environment based on real-time awareness features provided by the Software-Defined Networking (SDN) paradigm, assessing them against known vulnerabilities, and assigning them to a connectivity-appropriate network slice. The assessed entities are continuously monitored by an ML-based IDS, which is trained on an enhanced dataset. The experiment results showed that the adopted neural network, trained with heterogeneous data stemming from the operational environment (common vulnerability enumeration IDs that correlate attacks with existing vulnerabilities), can achieve more prediction accuracy than conventional one. The authors of [37] developed software vulnerability detection as an NLP problem with source code treated as texts, addressing the automated software vulnerability detection using recent DL NLP models. They compared various DL models based on their accuracy and the best performer achieved 95% of accuracy. Furthermore, the proposed approach was also able to predict the vulnerability class of source codes.

Recently, the Transformer-based architectures [38], such as BERT [39], were also leveraged in the CS domain, in particular for the definition of NER methodologies, able, among other things, to identify threats, vulnerabilities, and attacks mentioned in unstructured natural language texts. An example is the CyBERT model, presented by [40], which is able to implement a semi-automated CS vetting for Industrial Control Systems (ICS). This model was trained on a specifically created corpus of labeled sequences from ICS device documentation, collected across a wide range of vendors and devices, improving the obtained results compared to models trained on a generic domain. Furthermore, in [17], the author proposed a BERT-based model fine-tuned for the CS NER task, improving the obtained results using domain dictionaries. Another Transformer-based model presented in the literature is CyNER [41]. This model uses an XLM RoBERTa-large neural language model [42], pretrained on threat reports and fine-tuned for the NER task for the CS domain. Moreover, it also leverages further approaches to improve the NER results adopting a priority-based merging for extracting entities. In particular, it integrates regular expressions and KBs, a ML-based model for generic domain entities and a Flair-based [43] NER model. The authors of [16] presented a method for NER in the CS domain that uses a model that integrates BERT and BiLSTM-CRF DL architectures, improving baseline performance.

The next Table 1 summarizes the above reported works that focus on the Machine Learning models for the threat and vulnerability analysis, highlighting the adopted approaches and the corresponding advantages and limitations.

In summary, several works presented in the literature described ML-based techniques, specifically NLP approaches for the threat and vulnerability analysis but lack of focus on assessing the identified threats and vulnerabilities. Our work differs from these contributions not only because it specifically focuses on cyber attacks in the healthcare sector, but it leverages using NLP to extract relevant threats and vulnerabilities from the text and systematically assess them to determine the severity so that appropriate control measures can be taken into consideration.

## 3. Proposed Approach

The approaches for threat and vulnerability assessment described in the following paragraphs of this Section are part of an evidence-driven Risk and privacy Assessment methodology for Healthcare ecosystem (RA4Health), proposed within the AI4HEALTHSEC EC-funded H2020 project (https://www.ai4healthsec.eu (accessed on 30 October 2022)). AI4HEALTHSEC proposes a Dynamic and Self-Organized Artificial Swarm Intelligence Solution for Security and Privacy Threats in Healthcare ICT Infrastructures, which improves the detection and analysis of cyber attacks and threats on HCIIs, and increases the knowledge on the current cyber security and privacy risks. Additionally, AI4HEALTHSEC builds risk awareness, within the digital Healthcare ecosystem and among the involved Health operators, to enhance their insight into their Healthcare ICT infrastructures and provides them with capability to react in case of security and privacy breaches. Finally, AI4HEALTHSEC fosters the exchange of reliable and trusted incidents.

The RA4Health methodology aims to assist healthcare institutions to understand the associated individual and cascading risks, as well as to identify appropriate controls to mitigate the risks for a secure and resilient healthcare ICT infrastructure. RA4Health is the core of the AI4HEALTHSEC framework and includes the following five sequential phases:

- Determination of the Scope and Context;
- Analysis of the Health Care Supply Chain;
- Individual Risk Assessment;
- Cascading Risk Assessment;
- Risk Controls.

**Figure 1:**
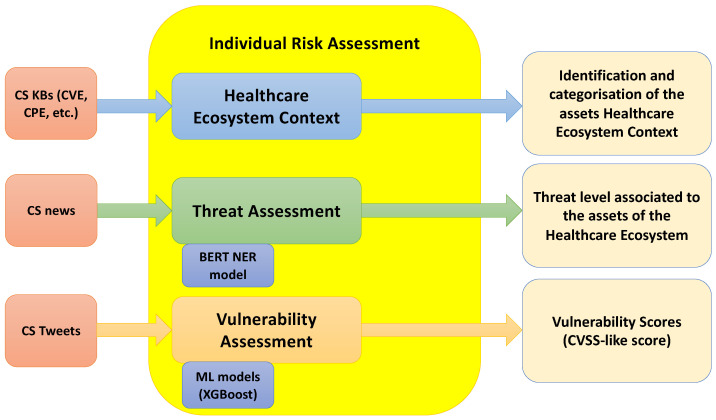
Individual Risk Assessment methodology schema.

The threat and vulnerability assessment methodologies presented in this paper are part of the third phase of the RA4Health methodology, namely the Individual Risk Assessment. The proposed methods aim to identify and assess the cyber threats and vulnerabilities for securing the healthcare ecosystem, by leveraging NLP approaches. The schema of the proposed individual risk assessment approach is depicted in the next Figure 1.

As shown in Figure 1, the Individual Risk Assessment methodology is formed by the following three main steps:

- *Healthcare Ecosystem Context*, which identifies the main assets of healthcare ecosystem context, including them into four distinct healthcare areas and categorizing them depending on their functionalities.
- *Threat Assessment*, which identifies and prioritizes the threats related to the services and assets of the HCII, adopting an NLP-based approach. The identified threats are categorized through threat taxonomies and then are assessed in a qualitative manner using threat scales.
- *Vulnerability Assessment*, which provides an automated vulnerability scoring system, based on a supervised ML solution.

The details of each step of the proposed approach are described in the following paragraphs of this Section.

### 3.1. Healthcare Ecosystem Context

A healthcare ecosystem is a complex system that consists of heterogeneous set of actors, entities, and systems (such as hospitals and social service organizations, medical equipment suppliers, pharmacies, health care research labs, devices developers, etc.) who are involved in the healthcare process and service delivery, including patient treatment, appointment, surgery and many others. This ecosystem is huge and includes a widely distributed network, including an interconnected set of healthcare entities (organizations, such as hospital agencies or clinics or individuals, such as doctors) that implement healthcare services which provision relies upon interdependent HCIIs (e.g., IT and Operational Technology (OT) systems) comprising interconnected sets of assets (e.g., implants, sensors, healthcare software, such as patients’ health records, pathology scanners and servers, medical X-ray equipment).

Within recent decades, there have been significant digital advancements within the whole ecosystem to support the healthcare service delivery and increase the interdependencies between physical and cyber levels. This composite and dynamic nature of digital interconnectivity has altered the threat landscape posing new cyber threats attracting the attention of adversaries to develop new security and privacy challenges committing sophisticated coordinated cyber-attacks that could cause a dramatic impact to the healthcare ecosystem. For instance, a cyber-attack on insecure imaging servers and unprotected data storages supporting medical x-rays can lead to the web exposure of sensitive information of patients, such as medical images and scans; or a comptonisation of a remote monitoring software of defibrillators could allow adversaries to take advantage of the system damaging the hospital equipment or amending of medical device configuration [8]. Therefore, it is necessary to identify and analyze the threats that could pose any potential risk within the ecosystem.

This step of the proposed approach investigates the overall healthcare ecosystem context based on the possible services and assets related to the services. Therefore, it includes service and asset inventory of the healthcare information infrastructure. A healthcare entity delivers various services and some of them are critical relating to patient treatment. It is necessary to generate a comprehensive list of services, e.g., patient appointment, remote consultation, surgery schedule, medical report, patient registration, etc. Service is viewed as a business process, where a collection of activities and tasks form a Business Flow, ensuring the proper operation of the service. Each business process is part of a specific healthcare ecosystem and may depend on external actors.

Once the services are identified, it is necessary to identify the assets which are related to them. Our approach advocates to use the Common Platform Enumeration (CPE) (https://nvd.nist.gov/products/cpe (accessed on 30 October 2022)) catalog to map the HCII assets with specific classes of applications, operating systems, and hardware devices. CPE provides a structure naming for the assets. The inventory tools and scanners can also assist to automatically identify the assets. The identified assets are the internal system components that are controlled by the examined healthcare organization(s). We have considered four distinct healthcare areas as presented in Table 2 to describe the assets within the HCII. For instance, in the area 1 are included infusion pumps, blood pressure monitors, insulin pumps, pace makers, heart rate sensors and other similar implants and sensors; the area 2 includes equipment such as ultrasound, MRI machines, electric hospital beds, workstation, healthcare management system, routers, etc.; services and processes such as appointment services, patient registration processes, diagnostic belongs to area 3; finally, clinics and hospitals are classified as interdependent HCIIs into area 4. Additionally, assets are also categorized depending on its functionalities, as shown in Table 3. This allows us to determine the importance of each asset within the ecosystem.

### 3.2. Threat Assessment

This step has the purposes of identifying and prioritizing the threats by following the services and assets. Individual threats can be considered as potential stepping stones to security risks (deliberate or accidental), which may affect those services and assets. The identified threats can be categorized through threat taxonomies and assessed in a qualitative manner using threat scales. The Threat Assessment includes two sub-tasks: it first performs a **threats identification** and then a **threats prioritization**. A preliminary threat assessment approach has been already presented in [44] and its evolution and improvements are described in this paper.

The threats identification task focuses on the potential threats for each asset of the HCII identified in the previous Healthcare Ecosystem Context step, exploiting threat intelligence data for this purpose. There are several available sources that catalog known threats along with their characteristics, such as Common Attack Pattern Enumeration and Classification (CAPEC) (https://capec.mitre.org (accessed on 30 October 2022)), to identify the threats relevant to the HCII. A set of threat characteristics from the CAPEC is considered to describe the threats. The full list of these characteristics is given below.

- *Abstraction*: Defines the different abstraction levels that apply to an attack pattern. A Meta level attack pattern provides an abstract characterisation of a specific methodology or technique used for an attack and generalization of a related group of standard level attack patterns. It is often void of specific technology or implementation and provides an understanding of a high-level approach.
- *Status*: Defines the different status values of an entry of the CAPEC catalog including view, category, attack pattern.
- *Description*: A short description of the threat.
- *Alternate Terms*: Indicates one or more other names used to describe this attack pattern.
- *Vendor and Item*: Respectively identify the vendor and item (e.g., *Google* and *Chrome*) affected by the CS issue.
- *Likelihood of Attack*: Determines the likelihood and severity of an attack that leverages using the attack pattern and may not be completely accurate for all attacks.
- *Typical Severity*: It is used to capture an overall average severity value for attacks that leverage this attack pattern with the understanding that it will not be completely accurate for all attacks.
- *Related Attack Patterns*: Refers to other attack patterns and related high-level categories. These relationships give insight to similar items that may exist at higher and lower levels of abstraction.
- *Execution Flow*: It is used to provide a detailed step-by-step flow performed by an adversary for a specific attack pattern. It is applicable to attack patterns with an abstraction level of details.
- *Prerequisites*: Indicates one or more prerequisite conditions necessary for an attack.
- *Skills and Resource Required*: Describe skill level or knowledge and possible resources (e.g., CPU cycles, IP addresses, tools) required by an adversary for an attack.
- *Indicators*: The possible indicators including activities, events, conditions, or behaviors that may indicate an attack which could be imminent, in progress, or has occurred. Each Indicator element provides a textual description of the indicator.
- *Consequences*: The possible consequences associated with an attack pattern. The required Scope element identifies the security property that is violated. The optional Impact element describes the technical impact that arises if an adversary succeeds in their attack.
- *Mitigation*: The suitable counter measure to prevent or mitigate the risk of an attack. The approaches described in each mitigation element should help improve the resiliency of the target system, reduce its attack surface, or reduce the impact of the attack if it is successful.
- *Example Instances*: It is used to describe one or more example instances of the attack pattern. An example helps the reader understand the nature, context, and variability of the attack in more practical and concrete terms.
- *Related Weaknesses*: Contains references to weaknesses associated with this attack pattern. The association implies a weakness that must exist for a given attack to be successful. If multiple weaknesses are associated with the attack pattern, then any of the weaknesses (but not necessarily all) may be present for the attack to be successful. Each related weakness is identified by a (Common Weakness Enumeration) CWE identifier (https://cwe.mitre.org (accessed on 30 October 2022)).
- *Taxonomy Mappings*: It is used to provide a mapping from an entry (Attack Pattern or Category) in CAPEC to an equivalent entry in a different taxonomy.
- *Notes*: It is used to provide any additional comments that cannot be captured using the other elements of the view.

**Figure 2:**
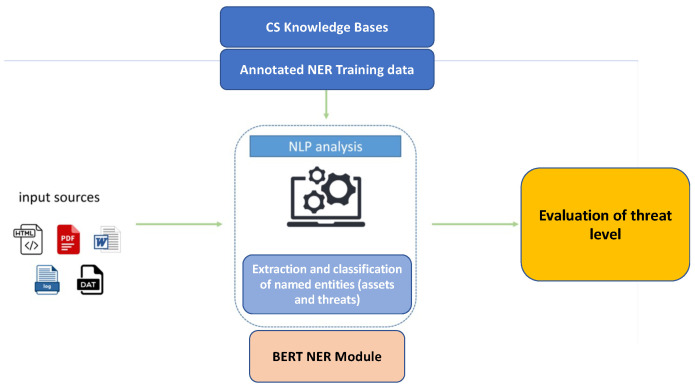
A conceptual schema of threat prioritization step.

The threats prioritization task allows the healthcare organizations to proactively determine the suitable controls to tackle the identified threats, providing an evaluation of the level of each identified threat. In particular, this task investigates threat-related information through a series of online available sources, ranging from CS news websites, CS blogs and social media, to threat and vulnerability catalogs for references of incidents related to specific CAPEC categories for the threat level calculation. For this purpose, we implemented an automated analysis of textual CS domain documents leveraging an NLP Named Entity Recognition (NER) approach, able to analyze unstructured NL textual documents in input, as presented in Figure 2. A set of input Natural Language sources corresponding for instance to threat reports, articles from various CS blogs/websites, Twitter data related to CS domain, online publicly available CS textual datasets, and/or log-files of the HCIIs can be fed into the NER NLP module. The NER module extracts assets and threats entity types, thanks to a previous training phase performed on a specific custom corpus, annotated with these classes of entities. Being a lack of annotated corpora in this domain, it is also necessary to annotate a specifically tailored NER corpus. For this purpose, we adopted a slight modification of the methodology presented in [45], which exploits both Distant Supervision (DS), Active Learning (AL) and a light human supervision, allowing annotating a NER dataset with a fraction of the effort required for a fully manual annotation.

The training corpus was used to fine-tune a BERT-based model [39] on the NER task. In particular, we adopted a BERT model pretrained on a large document collection belonging to the CS domain. The obtained fine-tuned NER model extracts the assets and the threats mentioned in natural language document collections. We adopted in our experiments a corpus of CS news extracted from the web, which is also periodically updated. The NER model can address the issues of a rule-based DS entity extraction, such as noisy or incomplete annotation, thanks to the generalization capabilities of the DL-based method [46], improving the detection of the relevant named entities.

The NER module is also leveraged to evaluate the level of the threats, which we correlate to the number of occurrences of each threat in the analyzed dataset. In particular, we calculate the percentage of the occurrence of each identified threat for an asset, increasing the number of the occurrence whenever the same threat and assets are mentioned in the same sentence. In this way, we assign a threat level based on this percentage of occurrence, as shown in Table 4. We assume that if the percentage of occurrence of a specific threat is high in the existing datasets, also its threat level is high. We identified five different levels, from *Very High* to *Very Low*.

### 3.3. Vulnerability Assessment

The last step has the purpose of building an HCII-oriented vulnerability exploit prediction scoring system. Therefore, we designed and implemented an automated vulnerability scoring system based on a supervised Machine Learning (ML) solution. Specifically, we exploit text data sources in order to train a supervised ML model, with the purpose of predicting the vulnerability score based on textual data, implementing in this way the vulnerability assessment step of the proposed risk assessment methodology.

**Figure 3:**
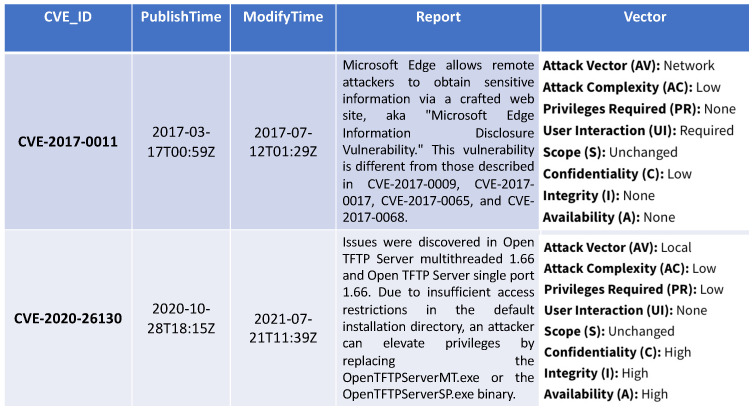
CVE data format.

We preliminarily investigate how a pool of CS-based text data can be used to assess a potential risk/vulnerability. For that reason, the proposed approach is built to estimate the vulnerability score based on CVE text data. Figure 3 presents in a tabular form the format of the used CVEs from NVD data. Each record corresponds to a specific CVE, associated with an id number (CVD_ID). The second and third column indicate the published and modified CVE incident time, respectively, while the fourth column contains a detailed report of each CVE incident. The last column corresponds to exploitability and impact metrics, namely the attack vector, attack complexity, privileges required, user interaction, scope, confidentiality impact, integrity impact and availability impact. Each row of the CVE KB is used to build a sample of our dataset, where the text is extracted from the *Report* column and the labels to train the supervised ML models correspond to the features listed in the *Vector* column.

**Figure 4:**
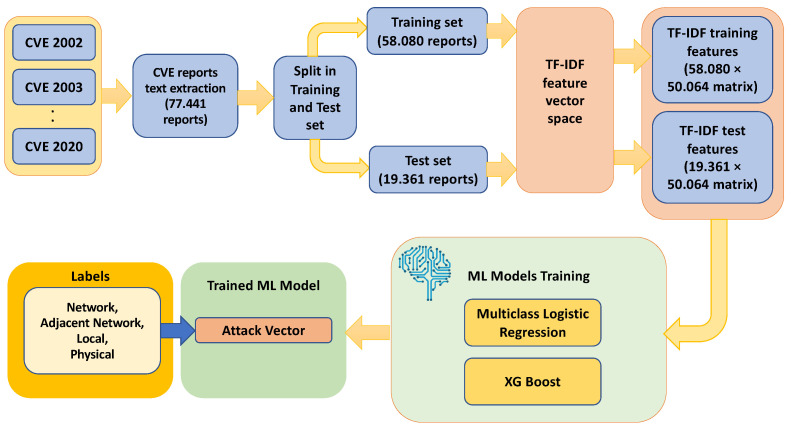
Flow diagram of the proposed supervised text-based machine leaning pipeline for the attack vector.

The process of estimating the vulnerability score invokes a supervised text-based ML model. Based on this, CVE reports from 2002 to 2020 were extracted to be used as a training dataset. Then, a Term Frequency Inverse Document frequency (TF-IDF) [47] is applied to obtain a numerical representation of the text data. Notably, TF-IDF is considered as a statistical measure that computes how relevant a word is to a document in a collection of documents. This is performed by multiplying two metrics, i.e., how many times a word appears in a document, and the inverse document frequency of the word across a set of documents. Figure 4 illustrates the data flow and data transformations and processes being invoked.

The first calculated metric corresponds to the Term Frequency TF of a word in a document TF(t,d), which corresponds to the number of times term (word) t appears in a document d. The Inverse Document Frequency IDF of the word IDF(t) across a set of documents corresponds to how common or rare a word is in the entire document set. Thus, the closer it is to 0, the more common a word is. IDF metric is computed by taking the total number of documents, dividing it by the number of documents that contain a word, and calculating the logarithm, and thus the TF-IDF formula is given by:

(1) $$TF-IDF=TF\left(t,d\right)+IDF\left(t\right)=TF\left(t,d\right)+\log\frac{1+n}{1+DF(d,t)}$$

where *n* denotes the number of documents and DF(d,t) is the Document Frequency of the term t. It is obvious that the higher the score, the more relevant that word is in that particular document. We also considered in the future works to test word embeddings [48] and FastText [49] features as numerical representation of the text, exploiting the large textual dataset extracted from CS news (described in Section 4.1) and collected to test the threat assessment step.

In the next stage, we trained a supervised ML model. In particular, we used a multiclass logistic regression model and an eXtreme Gradient machine (XGBoost) model [20] based on the computed training data, where the classes are the four values of the attack vector, namely:

- Network;
- Adjacent network;
- Local;
- Physical.

The trained model is validated on the test data and the test classification accuracy is finally estimated, as explained in detail in the next Section 4. We choose to adopt those two supervised ML models since the multiclass logistic regression model is considered as a simple to implement algorithm and can provide baseline results, while XGBoost is considered a modern, state-of-the-art algorithm, which can obtain higher accuracy within the described task.

**Figure 5:**
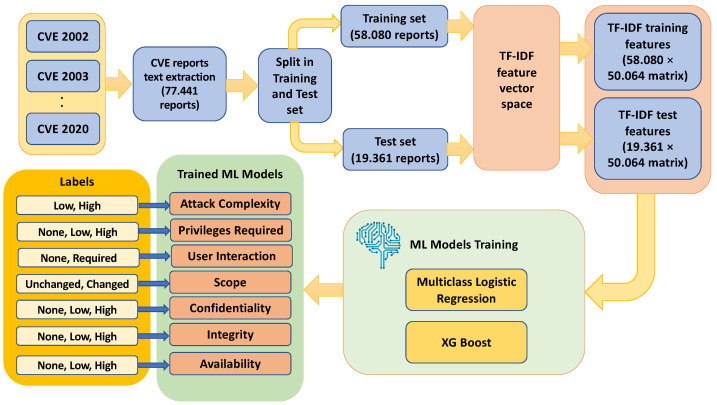
Flow diagram supervised text-based machine learning pipeline flow chart for exploitability and impact metrics.

The same process is followed for the rest of the impact and exploitability metrics, as shown in Figure 5. Specifically, we train a different supervised machine learning model for each different exploitability and impact metric, namely:

- Attack complexity (“low”, “high” labels);
- Privileges required (“none”, “low”, “high” labels)
- User interaction (“none”, “required” labels);
- scope (“unchanged”, “changed” labels);
- confidentiality (“high”, “low”, “none” labels);
- integrity (“high”, “low”, “none” labels);
- availability (“high”, “low”, “none” labels).

**Figure 6:**
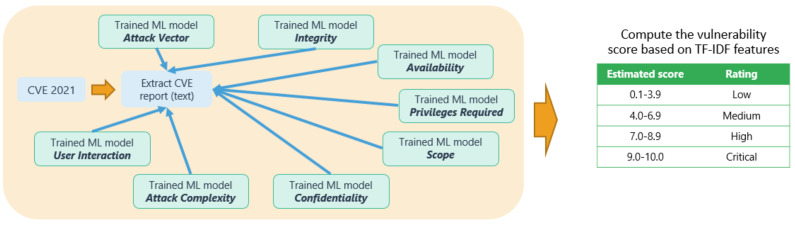
Unseen data evaluation phase.

At the end, we trained eight different ML models (multiclass classifiers), as shown in next Figure 6.

After the training of these eight different ML models, during the final out-of-sample (unseen data) evaluation phase we used the 2021 CVE reports, in order to estimate the vulnerability score. Figure 6 depicts the final unseen data evaluation phase, where the CVE reports of the year 2021 are extracted and transformed into numerical data representation via the TF-IDF procedure. Then, the eight different exploitability/impact metrics labels are estimated based on the eight different trained ML models. The estimated label of each exploitability/impact metric is exploited in order to compute a CVSS-like score with the value range shown in the green colored table of Figure 6. The CVSS-like score is computed based on the specification described in [50].

Since we are interested in evaluating the severity level of a potential vulnerability following the scale presented in the next Table 5, we finally perform the following data labelling based on the obtained CVSS-based score, which takes real values in the interval [0, 10], splitting this interval into five equal ranges, corresponding to a severity level which varies from *Very High*, in the case of CVSS-like score range from 8 to 10, to *Very Low*, in the case of CVSS-like score range from 0 to 2. Therefore, the task can be considered as a multi-class classification problem, with five classes being predicted implicitly via the estimated CVSS-like scores.

## 4. Experimental Assessment

This section describes the experiments we conducted for the threat and vulnerability assessment using the proposed methods. In detail, we first present the datasets, the used resources and tools and the metrics adopted to evaluate the results. Then, we describe the experiments, showing and discussing the obtained results.

### 4.1. Datasets

A CS news posts collection has been used for both fine-tuning and test the NER model of the Threat Assessment step, as well as for testing the threat level evaluation approach based on the occurrence of the threats and assets. This corpus has been extracted from The Hacker News website (https://thehackernews.com (accessed on 30 September 2022)), a CS news platform that attracts over eight million readers monthly, which is daily updated with the latest CS news and provides in-depth reports on current and future CS trends. The website contains tons of documents that describe threats, attacks, vulnerabilities and other CS topics. We developed a specific Python web scraper for this website, able to retrieve and collect the text from the news. The scraping task is performed weekly, to have a continuously growing and updated dataset. The dataset at the date of 6 September 2022 counts 514,220 tokens, extracted from 1065 news articles of the website.

This dataset has been also randomly split, to create an annotated dataset to train the NER model, and further split into a training and a test set. The remaining part of the dataset, called Threat Level (TL dataset), was used for the threat level assessment experiments, verifying the capability of the proposed approach to identify the threat level. The features of the whole dataset (called The Hacker News Dataset) and the other datasets obtained through a splitting of this data are summarized in the next Table 6, reporting the number of news posts in each dataset, the corresponding word count, the average number of words of the posts, the standard deviation of the word count, the total sentence count, the average sentence count and the standard deviation of the sentence count.

The text of the news has been extracted through a web crawler and a web scraper specifically implemented using a set of Python scripts. It is worth noting that the scripts run once a week, updating the dataset with the latest news, continuously increasing the available information for the future real-world applications of the proposed approach.

As explained in previous Section 3.2 the CAPEC and CVE KBs have been leveraged in the Threat Identification phase, allowing modeling assets and threats in the HCII, creating in detail a list of detected threats for each asset that operates for the provision of each identified healthcare service. Moreover, these two KBs were also used to support the annotation of the NER training set, by means of Distant Supervision (DS). The NER module is a supervised Deep Learning method, and so an annotated dataset is needed in order to train the model. Unfortunately, annotated NER datasets for the CS domain are not available and a custom dataset must be annotated for our purposes. We adopted an iterative hybrid Distant Supervision (DS) and Active Learning (AL) approach for the annotation of the CS NER training set proposed in [45], which leverages the knowledge extracted from these KBs in the DS phase. The CAPEC database used for the DS annotation of the NER datasets is structured as a JSON. It has been preprocessed, extracting the entries labeled as *threat*, their corresponding *product* and *vendor* labels in order to identify the assets, the description of the threats under the *description* label and the content of the *id* label, which include the coding of the corresponding threat (e.g., CVE-2021-37971, CAPEC-103, etc.). The relevant information has been included in a list, used to apply the DS for the annotation of the training set: the assets and their related threats mentioned in each sentence of the blog posts of the training set have been annotated be means of DS, after preprocessing the text by applying lowercasing, tokenization and sentence splitting.

The process of estimating the vulnerability score in the Vulnerability Assessment step invokes a supervised text-based ML model and leverages a different dataset, which is formed by CVE reports from 2002 to 2020 (see previous Figure 3). Considering that the total amount of these reports is 77,441, they were split in a training set and test set, respectively, randomly selecting the 75% and the 25% of the reports, leading to a training dataset of 58,080 reports and a test dataset of 19,361 reports, respectively. The summary of the features of the vulnerability dataset is reported in Table 7, showing the number of reports in each dataset, the corresponding word count, the average length of the reports, the standard deviation and the median.

**Figure 7:**
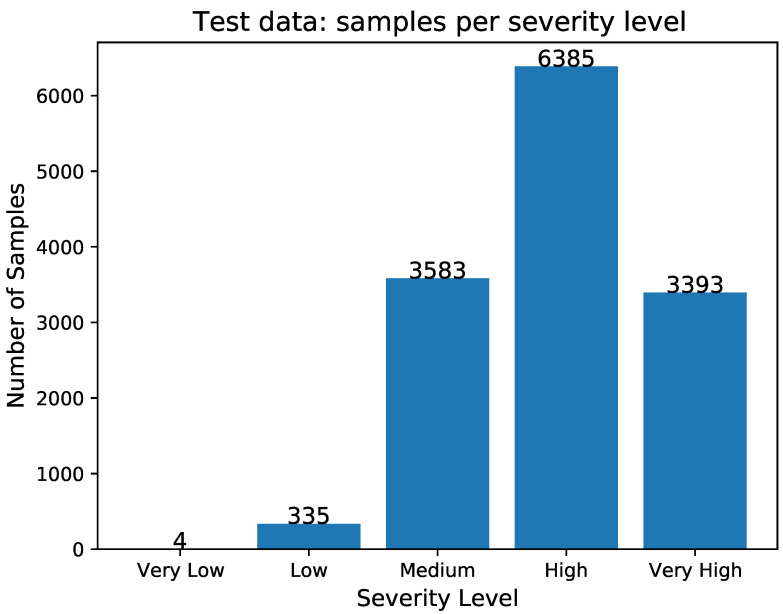
Histogram of the number of samples per severity level in the test data.

Figure 7 depicts the number of samples per severity level in the test data, and it is obvious that it is an unbalanced dataset. The details of the samples per severity level in the test data are also summarized in the next Table 8.

### 4.2. Resources and Tools

The NLP NER module of the threat assessment step relies on a BERT model pretrained on a very large CS document collection named SecBERT (https://github.com/jackaduma/SecBERT (accessed on 30 September 2022)). In detail, this model was pretrained on a corpus formed by: (i) APTnotes (https://github.com/aptnotes/data (accessed on 30 September 2022)), a collection of publicly available papers and blogs (sorted by year) related to malicious campaigns/activity/software that have been associated with vendor-defined APT (Advanced Persistent Threat) groups and/or tool-sets; (ii) the text extracted from the website included in Stucco-Data [51], a repository that keeps a list of the data sources that are potentially relevant to cyber security and the source for the web site to make the data sources easy to read (including the texts from CPE, CVE and other databases, as well as blogs, forums, bulletin boards, etc.); (iii) a corpus of corpus of 1000 English news articles from 2017 to 2019 used for the CASIE project [33]; (i) the datasets of SemEval 2018 Task 8 SecureNLP [52], a shared task on semantic extraction from CS reports. The model has 12 attention heads, 6 hidden layers and has an hidden size equal to 768. The SecBERT model has been fine-tuned on the NER task using the Huggingface Python library [53], which offers a set of API for training and fine-tuning Transformers-based Neural Language Models. The proposed NER approach can currently exploit any model included in the Huggingface library, such as RoBERTa-based models [42] and others. In our experimental assessment, we adopted SecBERT, which is based on classic BERT architecture, because it is shown in the literature that the pretraining on a closed-domain corpus is able to improve the performance of a neural language model when it is fine-tuned on tasks of the same domain [35,54].

For the preprocessing of the textual data and the implementation of the DS annotation of the dataset for the threat assessment experiments, we used Spacy [55], a flexible NLP Python library that includes tools for tokenization, sentence splitting and other NLP preprocessing tasks. The web scraper used to extract the news posts from the Hacker News web site has been implemented using Beautiful Soup Python library [56].

The ML models of the vulnerability assessment step were also implemented using Python libraries. In particular, the Logistic regression was implemented using scikit-learn Python library [57] (https://scikit-learn.org/stable/modules/generated/sklearn.linear_model.LogisticRegression.html (accessed on 20 September 2022)). The XGBoost model used the Dmlc XGBoost library [58], an optimized distributed gradient boosting library designed to be highly efficient, flexible and portable, which implements ML algorithms under the Gradient Boosting framework.

### 4.3. Metrics

The performance of the NER model was measured using the Precision (*P*), Recall (*R*), F1-Score (*F*1) and Accuracy (*Acc*) [59] metrics, defined as:

(2) $$P_{i}=\frac{tp_{c_{i}}}{tp_{c_{i}}+fp_{c_{i}}};$$

(3) $$R_{i}=\frac{tp_{c_{i}}}{tp_{c_{i}}+fn_{c_{i}}}$$

(4) $$F1_{i}=\frac{2\cdot P_{i}\cdot R_{i}}{P_{i}+R_{i}}$$

(5) $$Acc=\frac{\sum_{i=1}^{M}\frac{tp_{c_{i}}+tn_{c_{i}}}{tp_{c_{i}}+tn_{c_{i}}+fp_{c_{i}}+fn_{c_{i}}}}{M}$$

where $tp_{c_{i}}$, $tnc_{i}$, $fp_{c_{i}}$ and $fn_{c_{i}}$ are, respectively, the true positives, true negatives, false positives and false negatives for the class $c_{i}$, and *M* is the number of classes. $P_{i}$, $R_{i}$ and $F1_{i}$ are micro-averaged on all classes (threats and assets), obtaining *P*, *R* and F1 values reported in next Section 4.4.

The Mean Absolute Error (*MAE*), Mean Squared Error (*MSE*) [60] and R-Squared ($R^{2}$) [61], defined in the equations below, were used as performance metrics to evaluate the performance of the proposed automated vulnerability scoring system based on the two supervised machine learning models, namely multiclass logistic regression and XGBoost:

(6) $$MAE=\frac{1}{n}\sum_{i=1}^{n}\left|y_{i}-{\hat{y}}_{i}\right|$$

(7) $$MSE=\frac{1}{n}\sum_{i=1}^{n}{\left(y_{i}-{\hat{y}}_{i}\right)}^{2}$$

(8) $$R^{2}=1-\frac{\sum_{i=1}^{n}{\left(y_{i}-{\hat{y}}_{i}\right)}^{2}}{\sum_{i=1}^{n}{\left(y_{i}-\bar{y}\right)}^{2}},$$

where *n* is the number of the total scores, $y_{i}$ is the original vulnerability score of the i-th CVE incident, ${\hat{y}}_{i}$ is the predicted vulnerability score of the i-th CVE incident, and $\bar{y}=\left(1/n\right)\sum_{i=1}^{n}y_{i}$ based on the supervised Machine Learning pipeline described in previous Section 3.3.

### 4.4. Threat Assessment Experiments

The first part of the experiments aimed at testing and verifying the proposed threat assessment methodology. These experiments included a preliminary phase, where we tested the effectiveness of the NER model based on SecBERT, comparing the obtained performance in terms of Precision, Recall, F1-Score, and Accuracy [59] with (i) the ones obtained using DS and (ii) a baseline BERT model (*BERT-base-uncased* [39], pretrained on a large general-domain corpus) fine-tuned on the same training set. Then, the latter phase of the experimental assessment investigated the proposed threat prioritization approach, testing its capability to estimate the threat level from the NLP analysis of the CS news extracted from the web.

The results obtained from the preliminary experiments for the assessment of the NER model, in terms of Precision, Recall, F1-Score and Accuracy, are reported in the next Table 9. As we can see, the metrics confirm that the SecBERT model, pretrained on a large CS closed-domain corpora collection and fine-tuned on the dataset specifically created for our purposes, provides a slight performance boost, with respect to the baseline BERT model and a DS rule-based annotation.

The high Accuracy values have been obtained due to the very high number of true negatives $tn_{c_{i}}$, which is related to the dataset features (see previous Table 6), where the number of entities is very small if compared to the number of words that are not an entity.

The second part of the experimental assessment performed to investigate on the threat prioritization approach, testing the capability of the proposed methodology to evaluate the threat level. For this purpose, we applied the fine-tuned NER model to the Threat Level dataset. In this case, after a sentence splitting of the data, we extracted the mentions of threats and assets by means of the NER model.

The total number of assets and threats found in the TL dataset is reported in the following Table 10. Comparing this result with the number of news of the TL dataset (756), their average word count (478) and sentence count (14,595), reported in the previous Table 6, we can deduce that the NER module extracted at least one entity from more than the half of the sentences of the dataset. This results confirms that the adopted datasets contain a sufficient number of samples to test the threat assessment based on the proposed occurrence evaluation method, and, more in general, such kinds of datasets are a large information source, often not exploited.

After the extraction of the relevant entities, the same document collection has been preprocessed, applying sentence splitting, with the purposes of selecting only the sentences where a mention of both an asset and a threat is present, allowing in this way to identify the assets and the corresponding threats. In total, 2654 sentences containing a mention of both assets and threats were extracted. Example of these sentences, where the entities are in bold, and the corresponding class is indicated right after between square brackets, are reported below:

- *The**clipboard poisoning attack**[THREAT] is said to have been accidentally introduced in**Chrome version 104**[ASSET], according to developer Jeff Johnson.*
- *By uploading a JSP file to the**tomcat**’s [ASSET] root directory, it is possible to achieve**code execution**[THREAT], leading to**command execution**[THREAT].*
- *Threat actors are increasingly mimicking legitimate applications such as**Skype**[ASSET],**Adobe Reader**[ASSET], and**VLC Player**[ASSET] as a means to**abuse trust relationships**[THREAT] and increase the likelihood of a successful**social engineering attack**[THREAT].*
- *There are indications that**CVE-2021-22600**[THREAT] may be under limited, targeted exploitation,” Google noted in its**Android**[ASSET] Security Bulletin for May 2022.*

At this point, a threat occurrence table for each pair asset/threat mentioned in the same sentence is created through a custom Python script. This also allowed to calculate the corresponding percentages of occurrence of each pair, with respect to the whole dataset, defining in this way their respective level of threat, following the ranges of the percentage of occurrence shown in the previous Table 4.

Finally, it was possible to associate the threat level to the areas of the assets of the services of the HCIIs (summarized in the previous Table 2), previously identified by the Healthcare Ecosystem Context step. A mapping among those assets and the couples asset/threat extracted through NLP with the corresponding threat level has been performed, allowing for the identification of the threats of the HCII and the evaluation of their corresponding level. An example of the obtained results related to some of the founded assets is reported in Table 11, where some of the assets of the HCII and the corresponding threat levels obtained by the proposed methodology are shown.

As new data are obtained, the threat level identification task is relaunched and the percentage of assets/threats occurrences is updated, obtaining new percentages, as well as new assets and threats pairs. Moreover, the same approach could also be applied to different kinds of natural language datasets, formed by NL documents containing information related to assets and threats, such as CS social media posts, CS forums discussions and others. In this way, the threat level identification can rely on larger datasets.

The obtained results demonstrate that the SecBERT model, previously pretrained on CS document, can improve the CS NER performance, when this neural language model is fine-tuned on this task. Moreover, the application of the threat prioritization to the TL dataset showed that the proposed approach is able to identify a significant number of threats for a set of assets involved in the HCII, thanks to the previous Healthcare Ecosystem Context step, and to assign their corresponding threat level. This information can be exploited by the AI4HEALTHSEC CS situational awareness framework, supporting the monitoring and the prevention of CS incidents in the HCIIs.

### 4.5. Vulnerability Assessment Experiments

The purpose of these experiments was to assess the effectiveness of the vulnerability assessment methodology. We first applied the TF-IDF feature mapping to the CVE dataset described in Section 4.1, obtaining the corresponding feature representation of the textual data, which produced a training and test data matrix, respectively, of size 58,080 × 50,064 and 19,361 × 50,064.

As explained in Section 3, we adopted multiclass logistic regression and XGBoost as ML models. We performed a preliminary hyper-parameter tuning process, respectively, searching the following grids in the case of the logistic regression and XGBoost and selecting the best performing combination of settings:

- Logistic regression
  - penalty: [l1, l2]
  - C: [100, 10, 1.0, 0.1, 0.01]
  - solver: [liblinear]
  - max_iter: [100, 1000, 2500, 5000]
- XGBoost
  - n_estimators: [100, 400, 800]
  - max_depth: [3, 6, 9]
  - learning_rate: [0.05, 0.1, 0.20]
  - min_child_weight: [1, 10, 100]

As shown in Table 12, the obtained MAE, MSE and $R^{2}$ scores in the case of the multiclass logistic regression, respectively, equal to 0.9832, 2.8272, and −0.0333, while in the case of XGBoost, MAE, MSE and $R^{2}$ are, respectively, equal to 0.9326, 2.4744 and 0.0956. It is important to notice that the lower the error metrics MAE and MSE the better, whereas the higher the $R^{2}$ the better the predictive model is. It is experimentally confirmed that XGBoost performs better with the cost of higher computational complexity. It is also worth noting that here we do not want to perform a classic regression problem, but we apply these ML models to classify the level of each vulnerability metric, transforming them into a number using the CVSS-like score, predicting in this way the corresponding vulnerability levels.

**Figure 8:**
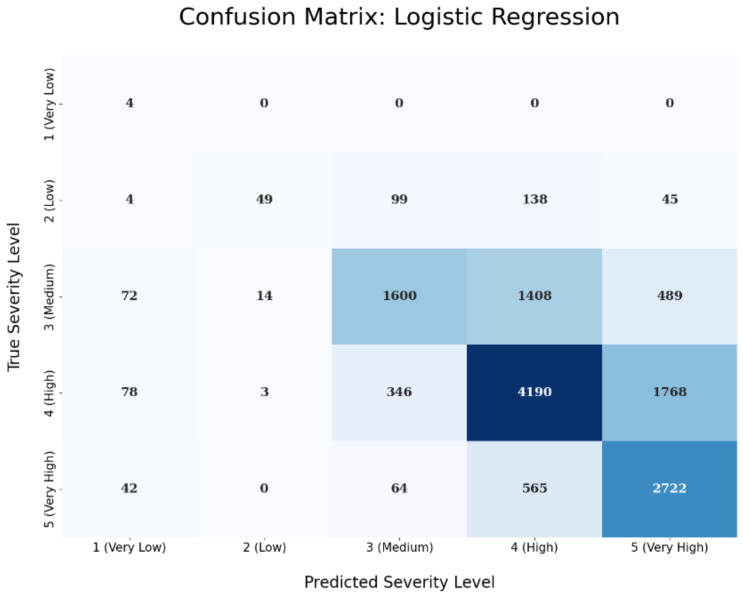
Confusion Matrix for Logistic Regression.

**Figure 9:**
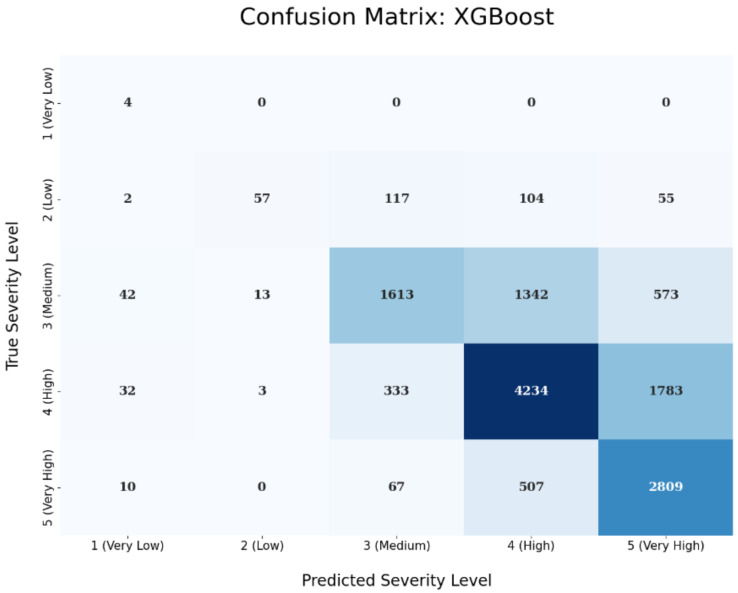
Confusion Matrix for XGBoost.

The confusion matrix of the logistic regression technique is depicted in the next Figure 8. The predicted severity levels are depicted in the x-axis, while on the y-axis the true severity levels are shows based on the test set’s samples. Ideally, a perfect predictive performance would result in a confusion matrix where we have values only on the diagonal, i.e., in a case where we classify correctly all the test samples for all the five different severity levels. The values in the boxes are just counts. For instance, in our case, the upper left box has a value 4 inside and the next four boxes have 0, 0, 0, and 0. This means that we are able to correctly classify all four ‘Very Low’ severity level test samples. On the other hand, by looking at the second row that refers to the ‘Low’ severity level, we can see that we classified correctly 49 ‘Low’ severity level test samples (out of the total of 335 that are in the test set) and missed 4, 99, 138, and 45 ‘Low’ test samples that were wrongly predicted as ‘Very Low’, ‘Medium’, ‘High’, and ‘Very High’ severity level, respectively. The same predictive performance interpretation holds for the rest of the confusion matrix rows and columns. Similarly, the confusion matrix of the XGBoost technique is presented in Figure 9.

**Figure 10:**
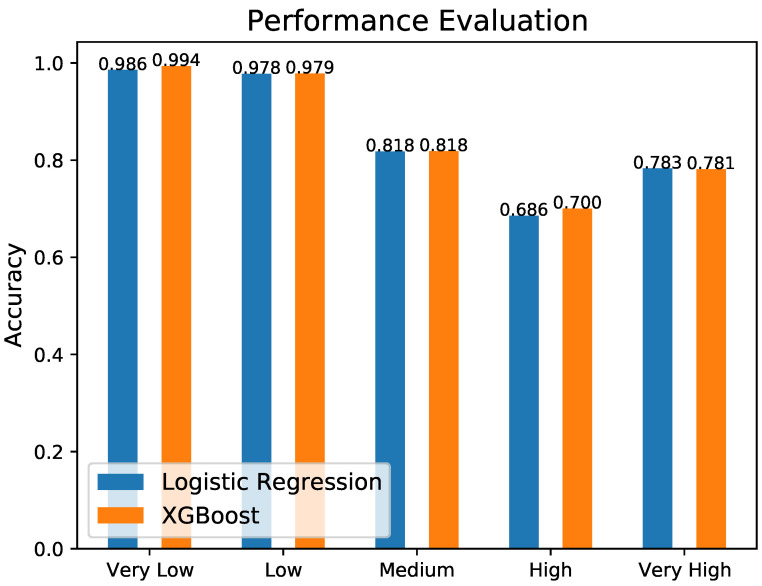
Comparison of Accuracy Level obtained with logistic regression and XGBoost.

Finally, Figure 10 shows the comparison of the obtained accuracy per severity level for the considered ML models. It is obvious that XGBoost performs, in terms of accuracy, slightly better than the logistic regression in all cases, apart from the ‘Very High’ case, where the logistic regression performed slightly better. Anyway, the analysis of the confusion matrixes in Figure 8 and Figure 9 demonstrates that the XGBoost obtains higher accuracy in all classes, confirming the overall better behavior of this ML algorithm.

## 5. Conclusions and Future Work

The paper presented a CS threat and vulnerability assessment methodology based on ML Natural Language Processing approaches, specifically developed with the purpose of securing the HCII and, more in general, of the whole healthcare ecosystem and its supply chains.

The proposed methodology includes three main steps. In the first one, the healthcare ecosystem context is modeled, identifying and categorizing its services and assets by exploiting Common Platform Enumeration (CPE) KB. Then, the potential threats for each asset of the HCII are identified using Common Attack Pattern Enumeration and Classification (CAPEC) KB.

The second step adopts a BERT-based neural language model fine-tuned on the CS Named Entity Recognition task, which extracts the mentions of the assets and threats within a Natural Language dataset composed of CS news posts extracted from the web, with the purpose of calculate the percentage of the occurrence of each extracted pair of threat/asset. In this way, it is possible to assign a threat level to each asset of the HCII identified in the previous phase, based on the obtained percentages.

Finally, the last step of the proposed methodology exploits ML logistic regression and XGBoost models to calculate a vulnerability score based on textual reports of vulnerabilities extracted from Common Vulnerabilities and Exposures (CVE) KB, adopting the Exploit Prediction Scoring System (EPSS) CVSS-like procedure.

The overall presented approach has the purpose of assisting the analysis of risks of the healthcare ecosystem, providing a level of threats and vulnerabilities related to the assets of the HCII, which can be used to determine the most appropriate controlling actions required to mitigate the risks. More importantly, the level of threats and vulnerabilities is obtained by the automatic analysis of natural language documents extracted from the web, allowing in this way to exploit this large and constantly updated information source.

The proposed methodology has been tested on two natural language document collections extracted from the web: (i) a set of CS news extracted from the Hacker News website, in the case of threat level assessment; and (ii) the textual reports included in the CVE KB from the year 2022, in the case of vulnerability level assessment. The obtained results demonstrated that the proposed method is able to automatically extract the required information and calculate the levels associated with the threats and vulnerabilities of the assets of the HCII. by analyzing natural language documents using ML models. Moreover, the performance of the adopted models obtained in the experimental assessment demonstrated that they can be integrated in real-world applications.

The method presented in this paper could be also further improved and tested as future work. For instance, the identification of the assets and threats of the HCII could be refined by applying Relation Extraction techniques [62], in order to better identify (and the corresponding pairs) and classify the relation between them, improving the calculation of their occurrence. We are also planning to test the proposed methodology on different datasets, including CS tweets, reports and other NL sources. In addition, the datasets used in this paper are constantly updated, by extracting the more recent news from The Hacker News site and the most recent reports from CVE, allowing the latest information to be available to update the calculations of the threat and vulnerability levels. Other planned tests include the adoption of the feature representation of the ML model of the Vulnerability Assessment step based on word embedding models, such as word2vec or FastText. These models can be trained on the available closed-domain CS corpora formed by the large document collections obtained within the development and experimental assessment of the proposed methodologies, improving the effectiveness of the vector spaces [35].

Finally, the proposed method will be integrated and tested in the next few months in real environments, within the pilot studies of the AI4HEALTHSEC H2020 EC-funded project.

## Figures and Tables

**Table 1:** Summary of ML models available in the literature for threat and vulnerability analysis.

Paper	Area	Method and Review
[29]	Review on ML and Data Mining techniques for software vulnerabilities	Vulnerability prediction based on text mining on software source code produced better result than metrics-based work despite availability of metrics. Anomaly detection approaches applicable with mature software system but lack of focus on security related vulnerabilities and high false positive.
[30]	Supply Chain threat analysis	Random Forest and XGBoost algorithm are used for the threat analysis with based on the threat intelligence features.
[31]	Identification of potential attack in smart healthcare system	Machine Learning and formal analysis capabilities are integrated for identification of attack vector based on Dynamic Casual Modeling (DCM) supervised and Automated decision-making (ADM) unsupervised ML model.
[32]	Cyber threat severity analysis	NLP based on logistic regression, used to identify the threat severity based on tweet data describing software vulnerability.
[15,18]	Cyber Security information/entity identification	NLP DL-based architecture is used for Named Entity Recognition (NER) in cyber security based on unstructured NER dataset. Data-driven DL with knowledge-driven dictionary method is used to improve NER.
[37]	Software code vulnerability detection	Automated software vulnerability detection using recent DL approaches. The vulnerability in software code is treated as an NLP problem.
[40]	Cyber Security Claim Classification	CS feature claims classifier based on BERT model, which also includes an approach to obtain optimal hyperparameters. The model obtains SOTA results, but it needs a specifically annotated corpus for the fine-tuning.
[17]	Cyber Security NER	A BERT-based model fine-tuned for the CS NER task. The obtained results are improved using CS-domain dictionaries.
[41]	Cyber Security NER	An XLM RoBERTa-large model pretrained on threat reports and fine-tuned for the NER task for the CS domain. The approach improves the performance by adopting other additional approaches (regular expressions and KBs, a ML-based model for generic domain entities and a Flair-based NER model), leveraging a priority-based merging for extracting entities.
[16]	Cyber Security NER	CS NER model that integrates BERT and BiLSTM-CRF architectures, improving baseline performance.

**Table 2:** Assets areas.

Area	Name
1	User interactions with implants and sensors
2	Medical equipment and IT devices
3	Services and processes
4	Interdependent HCIIs – Ecosystem

**Table 3:** Assets categories.

Category	Functionalities
Influence	Found in most organizations, distinct
Type	Software, hardware, Operating System (OS), information Sensitivity
Sensitivity	Restricted, unrestricted
Criticality	Essential, required, deferrable

**Table 4:** Threat level and corresponding percentage of occurrence in the dataset.

Threat Level	Percentage of Occurrence Range
Very High	[80–100]
High	[60–80]
Medium	[40–60]
Low	[20–40]
Very Low	[1–20]

**Table 5:** CVSS score with corresponding vulnerability level.

CVSS-Like Score Range	Severity Level
8.0,10	Very High
6.0,8.0	High
4.0,6.0	Medium
2.0,4.0	Low
0.0,2.0	Very Low

**Table 6:** Threat Assessment Datasets features.

Dataset	News Count	Word Count	Average Word Count	Word Stddev	Sentence Count	Average Sentence Count	Sentence Stddev
The Hacker News Dataset (6 September 2022)	1064	514,220	484.18	245.33	21,093	19.86	14.15
NER Training set	224	39,826	497.00	242.66	4708	21.11	13.75
NER Test set	84	20,086	490.87	205.28	1701	20.49	12.29
Threat Level (TL) dataset	756	454,308	477.91	247.92	14,595	19.36	14.35

**Table 7:** Features of CVE Reports used for vulnerability assessment.

Dataset	Reports Count	Total Word Count	Average Length	Standard Deviation	Median
CVE Dataset	77,441	2,880,401	37.19	15.34	34
Training set	58,080	2,153,576	37.08	14.63	34
Test set	19,361	726,785	37.54	17.51	34

**Table 8:** Number of samples per severity level in the vulnerability assessment test data.

Severity Level	Number of Samples
Very High	3393
High	6385
Medium	3583
Low	355
Very Low	4

**Table 9:** NER Results.

Method	Precision	Recall	F1-Score	Accuracy
DS	0.9554	0.7859	0.8623	0.9971
BERT	0.9569	0.7897	0.8654	0.9972
SecBERT	**0.9662**	**0.7995**	**0.8750**	**0.9975**

**Table 10:** Entities extracted in TL dataset.

Entity Type	Number of Entities
Threat	2145
Asset	6483

**Table 11:** Some examples of the threat level identified for some assets in the HCII.

Assets	Threats Level
Apache Tomcat	Medium
Adobe Reader	High
Google Chrome	Very High
Laravel framework	Low
Debian Linux	Medium
Android	High

**Table 12:** Logistic regression and XGBoost MAE, MSE and $R^{2}$ scores.

ML Model	MAE	MSE	$R^{2}$
Multiclass Logistic Regression	0.9832	2.8272	−0.0333
XGBoost	0.9326	2.4744	0.0956

## Data Availability

Not applicable.
